# Brief webcam test of hand movements predicts episodic memory, executive function, and working memory in a community sample of cognitively asymptomatic older adults

**DOI:** 10.1002/dad2.12520

**Published:** 2024-01-25

**Authors:** Renjie Li, Xinyi Wang, Katherine Lawler, Saurabh Garg, Rebecca J. St George, Aidan D. Bindoff, Larissa Bartlett, Eddy Roccati, Anna E. King, James C. Vickers, Quan Bai, Jane Alty

**Affiliations:** ^1^ Wicking Dementia Research and Education Centre University of Tasmania Hobart Tasmania Australia; ^2^ School of ICT University of Tasmania Hobart Tasmania Australia; ^3^ School of Allied Health Human Services and Sport La Trobe University Melbourne Victoria Australia; ^4^ School of Psychological Sciences University of Tasmania Hobart Tasmania Australia; ^5^ School of Medicine University of Tasmania Hobart Tasmania Australia; ^6^ Neurology Department Royal Hobart Hospital Hobart Tasmania Australia

**Keywords:** biomarkers, dementia, motor‐cognitive, preclinical, RapidMotionTrack, TAS Test

## Abstract

**INTRODUCTION:**

Low‐cost simple tests for preclinical Alzheimer's disease are a research priority. We evaluated whether remote unsupervised webcam recordings of finger‐tapping were associated with cognitive performance in older adults.

**METHODS:**

A total of 404 cognitively‐asymptomatic participants (64.6 [6.77] years; 70.8% female) completed 10‐second finger‐tapping tests (Tasmanian [TAS] Test) and cognitive tests (Cambridge Neuropsychological Test Automated Battery [CANTAB]) online at home. Regression models including hand movement features were compared with null models (comprising age, sex, and education level); change in Akaike Information Criterion greater than 2 (ΔAIC > 2) denoted statistical difference.

**RESULTS:**

Hand movement features improved prediction of episodic memory, executive function, and working memory scores (ΔAIC > 2). Dominant hand features outperformed nondominant hand features for episodic memory (ΔAIC = 2.5), executive function (ΔAIC = 4.8), and working memory (ΔAIC = 2.2).

**DISCUSSION:**

This brief webcam test improved prediction of cognitive performance compared to age, sex, and education. Finger‐tapping holds potential as a remote language‐agnostic screening tool to stratify community cohorts at risk for cognitive decline.

## 1 INTRODUCTION

Dementia prevalence is rapidly rising around the world and expected to reach 150 million by 2050.^1^ Alzheimer's disease (AD) accounts for more than 70% of cases, and the pathology begins 10–20 years before any overt cognitive symptoms emerge.^2^ Developing simple, low‐cost tests to detect this preclinical stage is a research priority for drug development and enriching cohorts for dementia prevention.^3^ However, we currently lack cost‐effective population‐level tests to risk stratify asymptomatic community samples; cognitive test performance is highly correlated with intelligence and education, and some people are more resilient to cognitive decline, carrying a higher burden of pathology for longer before cognitive test performance starts to decline.^4^ Positron emission tomography scans, cerebrospinal fluid (CSF) analysis, and blood‐based biomarkers are too costly, invasive, or specialist for widespread use.^5^

Emerging evidence indicates that analyzing hand motor function is a sensitive method to detect preclinical AD.^6^ Recent research (*n* = 72) has demonstrated that speed and rhythm of repetitive hand movements, measured by tapping on a computer keyboard, declined in cognitively healthy adults who had CSF AD biomarkers compared to those with normal biomarker levels.^7^ Relatively few studies have analyzed hand movements in AD and most required specialist wearable sensors or required researcher supervision.^8^, ^9^, ^10^ To harness the potential of hand movement analysis as a low‐cost population‐level test of preclinical AD, there remains a need to develop more accessible methods for remote administration.

Computer vision techniques can be applied to digital videos recorded through webcams in household computers or mobile phones to precisely measure movements.^11^, ^12^, ^13^, ^14^, ^15^ In contrast to using keyboard‐tapping tasks, video‐based technologies enable analysis of hand movements in 3D space (so additional features such as amplitude and decrement can be extracted), and the non‐touch technique minimizes infection risks. Computer vision analysis of finger‐tapping has not yet been employed via an online self‐test in home settings to aid prediction of cognitive performance in asymptomatic older adults.

In response to the urgent need for simple population‐level screening tests for dementia risk, we developed a home‐based online test, Tasmanian Test (TAS Test), that uses a computer webcam to record finger‐tapping videos, without requiring researcher assistance.^16^ Movement features from the videos are automatically extracted using computer vision algorithms that have been validated against wearable sensors.^17^ The objective of the study was to determine how well finger‐tapping movement features associate with cognitive performance in a community sample of cognitively‐asymptomatic older adults. Deficits in episodic memory are considered a proxy measure of preclinical AD as the hippocampus (critical for episodic memory function) is one of the earliest areas affected by AD pathology.^18^, ^19^ We thus hypothesized that (i) hand movement features predict cognitive performance over a model comprising age, sex, and level of education; (ii) nondominant hand movement features have stronger associations with cognitive performance than the dominant hand due to greater cognitive load required; and (iii) movement features associate more strongly with episodic memory than executive or working memory functions.^18^

## 2 METHODS

### 2.1 Study participants

Participants were recruited from the Island Study Linking Aging and Neurodegenerative Disease (ISLAND) project, a 10‐year public health initiative that launched in 2019 at the University of Tasmania, Australia; the detailed protocol has previously been described.^20^ In brief, the project recruits people aged 50 years or older who live in the Australian state of Tasmania and aims to reduce their dementia risk through education on modifying lifestyle and medical factors.^21^ ISLAND participants who had completed their annual research surveys were invited to the TAS Test sub‐study. TAS Test is an online battery of motor‐cognitive tasks, designed to be completed on a desktop or laptop computer without researcher assistance. The detailed TAS Test protocol has been described by Alty et al.^16^; in brief, participants log into a website, provide online consent, and are guided through a series of tasks, including video‐recorded assessments of finger‐tapping.

**RESEARCH IN CONTEXT**

- **Systematic review**: The authors reviewed journal articles and abstracts using PubMed and Google Scholar. Emerging evidence suggests hand movement analysis holds strong potential to identify people at risk of Alzheimer's disease. Relevant citations are cited.
- **Interpretation**: A total of 404 cognitively asymptomatic adults aged 51–84 completed self‐administered 10‐second‐finger‐tapping tasks at home via the TAS Test website, recorded via a webcam. Hand motor features improved prediction of episodic memory, executive function and working memory. Dominant hand features were more predictive for cognitive function, than nondominant hand features.
- **Future directions**: Brief self‐administered online hand movement tests using a webcam may aid identification of pre‐symptomatic cognitive performance. This provides a low‐cost accessible and language‐agnostic method for stratification of enriched cohorts for further assessment. Future work should compare finger‐tapping webcam tests to other validated biomarkers of preclinical Alzheimer's disease. Prospective longitudinal designs are required to support the association between hand motor performance and cognitive function.

### 2.2 Data collection

#### 2.2.1 Demographic and clinical details

Participants provided information on their age, sex, and hand dominance (right/left/ambidextrous) via the TAS Test website. As part of the ISLAND project, they completed questionnaires on their level of education (left formal education before 16 years old/at age 16/ at age 17–18, undergraduate degree or equivalent, Master's degree or equivalent, and PhD or equivalent) and medical diagnoses (including dementia, AD, multiple sclerosis, and Parkinson's disease). They also responded to the following three questions about cognitive symptoms: “Have you noticed a substantial change in your memory and mental function in recent years?” “Have you been told by a doctor that you have dementia?” “Have you been told by your doctor that you have a memory impairment, but they were uncertain if you have dementia?”. If participants responded “yes” to any of these questions, they were excluded from further analysis as this study focused on assessing motor predictions of cognitive performance in those *without* overt cognitive problems. Participants with Parkinson's disease and multiple sclerosis were also excluded.

#### 2.2.2 Finger‐tapping data collection

**FIGURE 1:**
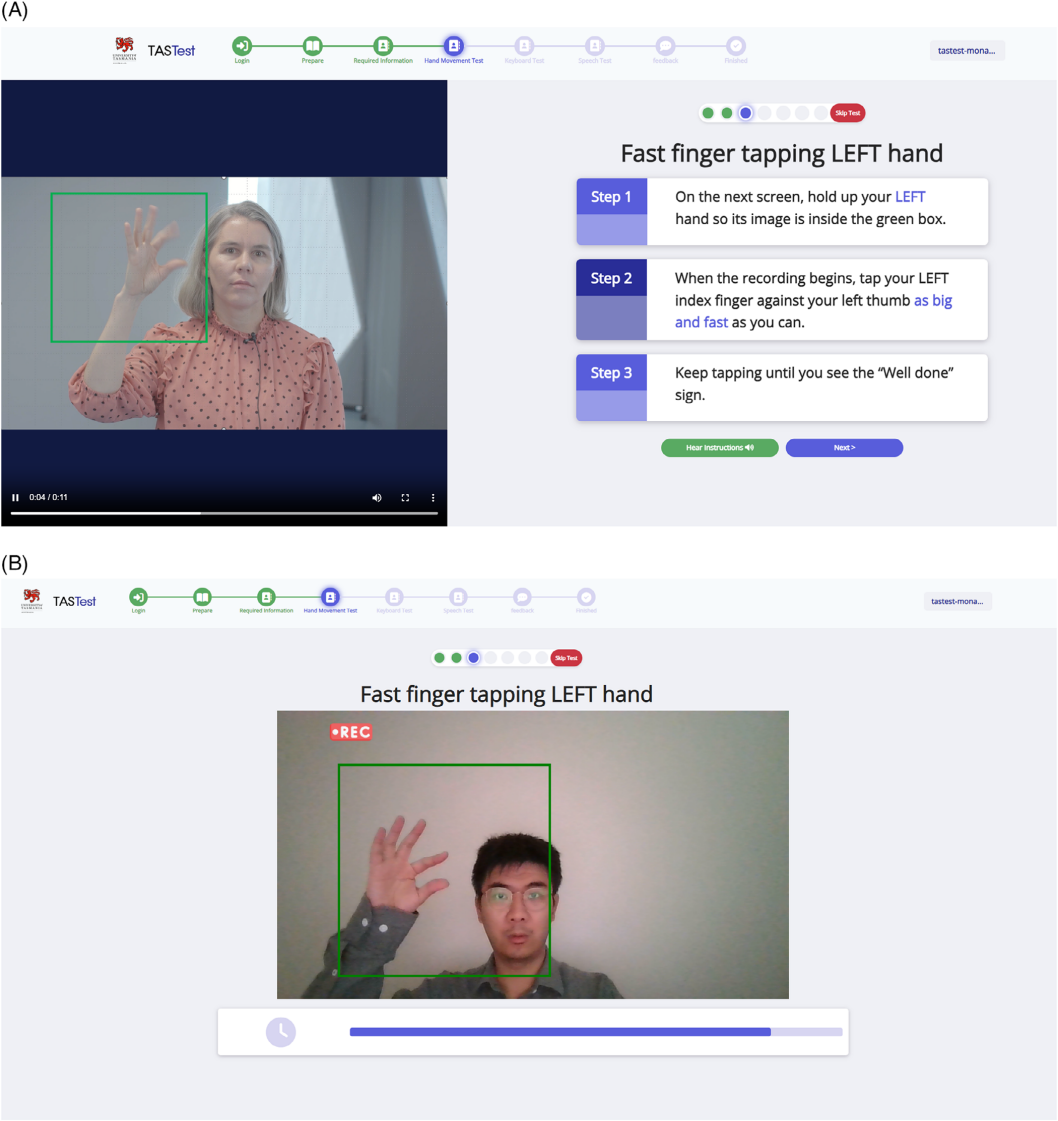
Screenshots of TAS Test finger‐tapping tests with green boxes guiding the participants where to position their hands for good quality data recording; (A) instruction screen, (B) recording screen (mirror view from the participant's perspective)

Participants completed TAS Test finger‐tapping tasks remotely as a “self‐test” using their own computer.^16^ TAS Test began with a series of general instruction screens to help participants ensure their video camera was working and to guide correct positioning of their hands. For each finger‐tapping test, there was an instruction screen followed by a recording screen. On the former, a looped video played automatically to demonstrate the task, with written instructions next to it; the participant could press an “audio icon” to also hear the instructions. For each finger‐tapping task, the participant was instructed to hold their hands about 50 cm from the computer camera so they could see them fitting inside green‐outlined data collection regions that appeared on the screen, and then to “Tap your index finger against your thumb as big and fast as you can” (Figure 1A). The participants decided when they were ready to record by pressing “Next” to move to the recording screen.

On the recording screen, a 5‐second visible countdown automatically started to allow time for the participant to position their hands correctly. This was followed by a 10‐second recording period, signaled by a flashing red circle on the side of the screen with the word “recording” (Figure 1B ). Participants tapped their index finger against their thumb until the recording stopped, indicated by a message on the screen that stated, “Well done, you have completed the task”. Finger‐tapping of the right hand was recorded first, followed by the left. The video recordings of hand movements were automatically timestamped and saved as an MP4 file to the TAS Test database.

#### 2.2.3 Movement data extraction from finger‐tapping videos

We applied validated deep learning‐based computer vision algorithms called “RapidMotionTrack”, or RMT,^17^ to automatically extract movement features from the finger‐tapping videos. RMT outperforms other state‐of‐the‐art, computer vision methods^17^, ^22^ in measuring finger‐tapping features and has been validated against gold‐standard Optotrak wearable sensor systems, that record movement in 3D space 250 times per second.^17^ The movement features extracted by RMT comprised mean tapping frequency (M_TF_), total tapping count (TTC), mean inter‐tap interval (M_ITI_), maximum speed (S_MAX_), coefficient of variance of tapping frequency (COV_TF_), intra‐individual variability (IIV), and coefficient of variance of amplitude (COV_A_).^17^

### 2.3 Cognitive performance

The Cambridge Neuropsychological Test Automated Battery (CANTAB) measures cognitive performance and includes online versions which are culturally neutral and have been validated against in‐person neuropsychological assessments.^23^ Participants completed two CANTAB tests: Paired Associates Learning (PAL), a test of visual episodic memory, and Spatial Working Memory (SWM), a test of executive function and working memory.

#### 2.3.1 Episodic memory

**FIGURE 2:**
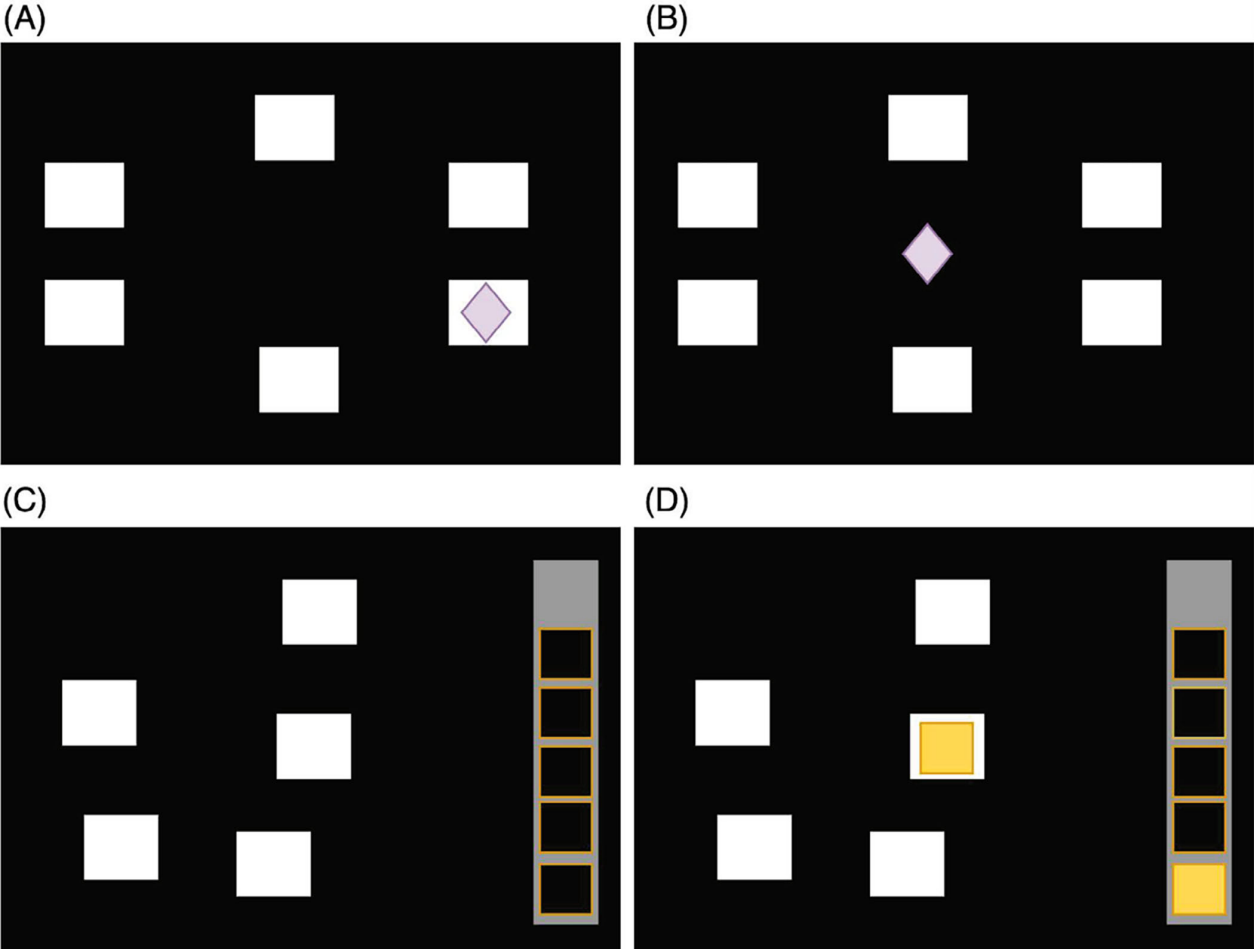
A schematic diagram of the stages of the Paired Associates Learning (PAL) and Spatial Working Memory (SWM) cognitive tests. For PAL, (A) participants were required to remember different patterns “stored” in different boxes on the screen. Boxes “opened” randomly and one or more contained a pattern. (B) A new pattern would appear at the center of the screen, and participants were asked to select the box where the same pattern was stored. For SWM, (C) and (D) participants were required to click on the boxes where yellow tokens were stored. Yellow tokens could only appear once in one box per attempt. In each attempt, there could be three, four, or six boxes which had yellow tokens.

Participants completed PAL, which typically takes about 8 minutes. First, they were required to remember different patterns “stored” in boxes on the screen. Second, boxes “opened” randomly and one or more contained a pattern (Figure 2A). Third, a new pattern appeared at the center of the screen, and participants were asked to select the box where the same pattern was stored (Figure 2B). As participants progressed through the stages, the number of new patterns increased from one to eight. The PALTEA6 score is the total errors at the six‐pattern stage, adjusted for incomplete or failed trials; it was used in the present study as it is widely recognized as a sensitive measure of cognitive deficits in memory and learning abilities.^24^

#### 2.3.2 Working memory and executive function

SWM assesses key aspects of working memory and executive function and typically takes around 5 minutes. Participants were required to click on boxes one by one to “open” them and find out where yellow tokens were stored (Figure 2C,D). In a single attempt, a yellow token was restricted to appearing only once in a single box. The number of boxes that contained a yellow token in each attempt could be either 3, 4, or 6. The number of times a participant re‐clicked a box that they had already opened earlier in the same attempt is called the “Between Errors Score”. The “SWMBE6”, which refers to the Between Errors Score adjusted for the six tokens stage, was used as a validated measure of spatial working memory. The Spatial Working Memory Strategy score (SWMS), defined as the number of distinct boxes used to begin each new search, is a measure of executive function (planning and decision‐making).^23^

### 2.4 Data analysis

The different movement features extracted were transformations of the same video data, and thus we must expect multicollinearity (e.g., frequency and amplitude may be collinear because larger movements take longer to execute for a given average velocity) along with the attendant concerns about how coefficients and *p*‐values should be interpreted in the context of multiple regression.^25^ To address our hypotheses, regarding whether hand movement features would predict cognitive test performance over and above demographic variables, we adopted a model selection by penalized likelihood approach where a parsimonious model is sought rather than an attempt to identify single features with significant *p*‐values. We used Akaike Information Criterion (AIC)^26^ with a penalty of 2 to compare how different models of finger‐tapping parameters predicted PALTEA6 and SWM scores, where a lower AIC denotes a better predictive model, penalized for the number of terms to favor parsimony and avoid overfitting (where a higher unpenalized likelihood occurs simply because more features tend to explain more variance in the fitted data, but not necessarily in new data). A better predictive model with finger‐tapping features included can be interpreted as evidence of construct validity^27^; that is, at‐home recording of finger‐tapping can predict cognitive function (adjusted for age, sex, and education).

Cognitive test scores were counts so we applied generalized linear models, assuming residuals followed a negative binomial or Poisson distribution (whichever had the lower AIC). We compared a null regression model (Model_null_) of variables comprising age, sex, and level of education to a series of models that comprised these same variables *plus* different combinations of finger‐tapping movement features (${\mathrm{Model}}_{i},\;i\in I$, where I is the set of every linear, additive combination of finger‐tapping measures). This was implemented with the “dredge” function from the “MuMIn” R package.^28^ The purpose of the null model is simply to capture the amount of variance which can be explained using readily captured demographic data which are known to be associated with cognitive task performance. If Model_i_’s AIC was more than 2 units lower than Model_null_’s AIC (ΔAIC > 2), Model_i_ and Model_null_ were considered statistically different. Therefore, the linear combination of finger‐tapping measures in any Model_i_ with ΔAIC > 2 indicates those motor features improve prediction of cognitive performance over and above the null model. Models with ΔAIC < 2 are considered statistically equivalent. ΔAIC statistics reported in‐text is for the best‐ranked model (i.e., model with lowest AIC) unless otherwise noted. We report Nagelkerke's adjusted *R* ^2^ for proportion of variance explained. We also fitted Random Forest regression models using the randomForest R package^29^ and reported RMSE and variable importance statistics in Supplementary materials for comparison.

## 3 RESULTS

A total of 680 participants completed CANTAB assessments and finger‐tapping tasks; however, after removing participants who did not provide video of sufficient quality for both hands, there were 404 participants, with mean (SD) age of 64.6 (6.74) years (70.8% female). Table 1 outlines demographic data and CANTAB cognitive test scores. Common reasons for videos being of insufficient quality were malposition of the hands outside the camera frame and late commencement of finger‐tapping after the 5‐second countdown.

**TABLE 1:** Demographic details of the included participants

Parameter	*N* = 404
**Age (years)**	
Mean (SD)	64.6 (6.74)
Median [Min, Max]	64.0 (51.0, 84.0)
**Gender**	
Female	286 (70.8%)
Male	118 (29.2%)
**Educational attainment**	
Left formal education before 16 years old	15 (3.7%)
Left formal education at age 16	30 (7.4%)
Left formal education at age 17–18	51 (12.6%)
Undergraduate degree or equivalent	201 (49.8%)
Master's degree or equivalent	81 (20.0%)
PhD or equivalent	26 (6.4%)
**PAL (total errors six‐pattern adjusted)**	
Mean (SD)	3.96 (4.30)
Median (Min, Max)	3.00 (0, 22.0)
**SWM (between errors six‐pattern)**	
Mean (SD)	2.93 (3.28)
Median (Min, Max)	2.00 (0, 13.0)
**SWM (strategy)**	
Mean (SD)	7.54 (2.80)
Median (Min, Max)	8.00 (0, 13.0)

Abbreviations: PAL, Paired Associates Learning; SD, standard deviation; SWM, Spatial Working Memory.

### 3.1 Hand movement features improved prediction of cognitive performance

**FIGURE 3:**
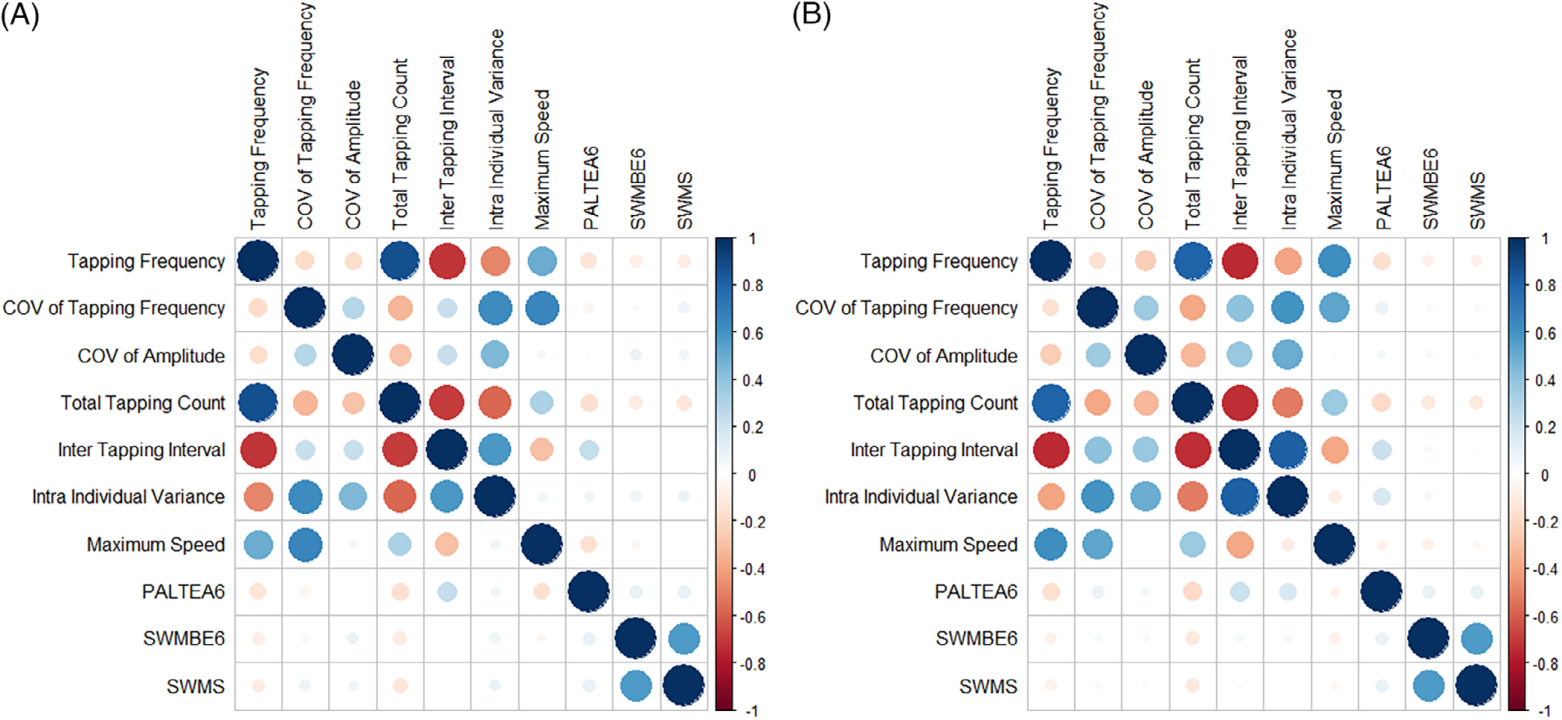
Correlation matrix of dominant (A) and nondominant (B) hand finger‐tapping features. The size of the circles denotes the strength of correlations between different features. The intensity of the shading color denotes the strength and direction of correlations with red shades representing negative correlations and blue shades representing positive correlations

Dominant hand and nondominant hand finger‐tapping features improved model fit significantly (compared to the covariate‐only null model) for all cognitive tests, except for nondominant hand features in predicting working memory test scores (SWMBE6). Table 2 details AIC for the null model, the best ranked (by AIC) finger‐tapping model, and percentage variance explained for each of the cognitive test measures of episodic memory (PALTEA6), working memory (SWMBE6), and executive function (SWMS). Figure 3 shows the correlation matrix of different movement features with PALTEA6, SWMBE6, and SWMS.

**TABLE 2:** Akaike Information Criterion (AIC) and adjusted *R* ^2^ for covariate‐only null model and best ranked model with finger‐tapping features included

Cognitive domain (test)	Hand	AIC_null_	AIC_best_	ΔAIC	Adj‐*R* ^2^_null_	Adj‐*R* ^2^_best_
Episodic memory (PALTEA6)	Dominant	2004.7	1998.3	6.4	9.9%	12.8%
	Nondominant	2004.7	2000.8	3.9	9.9%	11.9%
Executive function (SWMS)	Dominant	1984.7	1977.5	7.2	14.3%	17.2%
	Nondominant	1984.7	1982.3	2.4	14.3%	15.7%
Working memory (SWMBE6)	Dominant	1770.5	1767.4	3.1	4.7%	7.4%
	Nondominant	1770.5	1769.6	0.9	4.7%	5.8%

Abbreviations: AIC, Akaike Information Criterion.

For episodic memory, all hand movement features appeared in the ΔAIC > 2 set except for M_TF_ and COV_A_ in the dominant hand, and M_TF_, TTC, and M_ITI_ in the nondominant hand. For executive function, all hand movement features appeared in the ΔAIC > 2 set except for M_TF_, in the dominant hand, and M_TF_, *S*_max_ and COV_A_ in the nondominant hand. For working memory, all dominant hand movement features, and all nondominant hand movement features, appeared in the ΔAIC > 2 set except for COV_A_ in the nondominant hand.

### 3.2 Dominant hand movement features outperformed the nondominant hand

Movement features from the dominant hand were significantly better than those from the nondominant hand at predicting cognitive test scores across each of the three cognitive domains ΔAIC = 2.5 (episodic memory), ΔAIC = 4.8 (executive function), and ΔAIC = 2.2 (working memory).

### 3.3 Movement features better predicted executive function than memory

Movement features were associated more strongly with executive function than episodic memory or working memory functions; Table 2. Using the dominant hand movement features, the best‐ranked model had ΔAIC 7.2 ($R_{\mathrm{adj}}^{2}$ = 17.2%) for executive function, compared to ΔAIC 6.4 ($R_{\mathrm{adj}}^{2}$ = 12.8%) for episodic memory and ΔAIC 3.1 ($R_{\mathrm{adj}}^{2}$ of 7.4%) for working memory.

## 4 DISCUSSION

Our findings supported *the first hypothesis* – hand movement features improved prediction of cognitive performance across tests of episodic memory, executive function, and working memory over and above models which included age, sex, and level of education. The findings did not support *the second hypothesis*; according to Table 2, movement features from the dominant hand were significantly better at estimating cognitive performance than the nondominant hand. *The third hypothesis* was also not supported: movement features associated more strongly with executive function than episodic memory or working memory functions.

This is the largest study examining finger‐tapping and cognitive function (*n* = 404), the first to evaluate finger‐tapping with individual cognitive domains, and the first to use a webcam‐based self‐test. Only five previous studies (total *n* = 310 participants, including largest study *n* = 102)^30^ examined finger‐tapping in dementia or mild cognitive impairment (MCI); these generally reported slower speed and impaired rhythm, with 4 using wearable sensors and one a subjective clinical rating.^31^

Key‐tapping is similar to finger‐tapping as it involves the index finger pressing a target (e.g., a pressure transducer or computer key) but these studies lack amplitude measures or relative measures to the thumb. A meta‐analysis included three key‐tapping studies in MCI and early AD (*n* = 254) and found movement features had a pooled sensitivity/specificity of 0.85/0.82 for discriminating MCI/AD dementia from healthy controls (HCs).^32^ Two studies examined cognitively‐asymptomatic adults’ key‐tapping performance in association with AD biomarkers – Mollica et al. found slowed and less regular tapping in preclinical AD (CSF‐biomarker defined) compared to controls (*n* = 72).^7^ Wang et al. examined key‐tapping in 1169 asymptomatic older adults (using PALTEA6 as a proxy measure of preclinical AD) and found that speed, variability, dwell time, and inaccuracy scores improved predictions of episodic memory and executive function, but not of working memory.^33^ Both studies reported motor variability in the best‐performing models, which aligns with the present study's findings of COV_TF_, M_ITI_, and IIV (measures of rhythm).

The present study's finger‐tapping results generally align with previous finger‐ and key‐tapping studies that report reduced frequency and increased variability characterize cognitive impairment across the dementia continuum.^7^, ^31^, ^34^, ^35^, ^36^, ^37^Together, these studies suggest that the ability to maintain a fast‐ and regular‐paced hand motor rhythm is associated with cognition, especially episodic memory and executive function. They also align with gait studies that report walking speed, and step variability, associate most strongly with memory and executive function.^38^

The neural mechanisms for these motor‐cognitive associations remain uncertain. Most studies analyzed gait dysfunction as it is well‐established to predict incident dementia^39^ and precedes cognitive decline^6^; these reported slower speeds associated with higher amyloid burden,^40^ smaller hippocampal volume^41^ ^,^ and prefrontal deactivation. Koppelmans et al. examined neural biomarkers of key‐tapping tests in MCI (*n* = 27), AD (*n* = 39), and HC (*n* = 47) and found hand motor performance associated with hippocampal volume but not with *apolipoprotein E* (*APOE)* *ɛ*4 alleles or global amyloid‐β deposition, leading the authors to conclude that later stage AD pathology is associated with motor changes.^34^ However, this contrasts with a recent imaging study (*n* = 601, HC, and AD) that found decreased segregation of brain networks occurs early in AD and is more widespread than expected – involving not only cognitive association areas, but also sensory and motor regions.^42^ Notably, the AD‐related network alterations were independent of amyloid pathology and *APOE* *ɛ*4 status, and distinct from aging‐related functional network alternations that usually spare sensory‐motor systems relative to association systems.^43^

The strengths and limitations of this work are acknowledged. In terms of strengths, we examined a wide range of ages from a community cohort, analyzed each hand separately, evaluated movement components in detail, and used validated cognitive tests across different domains. We also adjusted for covariates known to be associated with cognitive performance. Further, TAS Test finger‐tapping tests are brief simple self‐tests, free to use at no cost, whereas CANTAB charges for both cognitive tests, and the test duration of 20 s recordings compare well to 13 minutes for CANTAB.

The use of computer vision methods to extract finger‐tapping features to predict *cognitive* performance is novel^3^, ^5^, ^16^ and efficient^12^ but similar methods are increasingly being used to quantify various *movement* disorders as they are objective and granular, unlike clinical rating scales,^44^ and provide evaluation of movements in 3D space through simple smartphones or laptops.^14^, ^15^, ^45^ Computer vision approaches facilitate wide reach and accessibility, including to those in rural and remote regions, as webcams are so ubiquitous in phones and computers around the world. Furthermore, advanced computer vision techniques ensure that home‐based self‐testing is possible as movement features can reliably be extracted despite real‐life cluttered backgrounds^13^ and blurred images due to low‐level lighting or low frame rate webcams.^46^

We acknowledge that with home‐based tests, there is an increased risk of poor data collection; approximately one‐third of participants did not provide videos of *both* hands. Extending the findings of the study to future work, measuring just a single hand (the dominant hand) may rectify this issue. Second, we acknowledge that participants are not representative of the broader community, as the majority were women, had healthy bias, were relatively well‐educated, and of White, Northern‐European ancestry. We recognize, as a cross‐sectional study, we cannot examine cognitive decline. There will be unmeasured variables that may affect motor function too. We also acknowledge that hand dominance was determined by a single question rather than a validated handedness questionnaire. Given the nature of the study and the complexity of cognitive testing, it is reasonable to expect a relatively low $R_{\mathrm{adj}}^{2}$. The intention is not to claim that hand movement features explain a large proportion of the variance in cognitive scores. Instead, we aim to demonstrate that there is an association between finger‐tapping and cognitive test performance after adjustment for confounding, supporting our hypothesis and indicating that these motor features are associated with cognitive functions including memory and executive function.

Future work should explore a wider array of finger‐tapping tasks (bilateral, anti‐phase, dual task, etc.), or different combinations of hand movement features, to predict a wider range of cognitive domain scores. With larger studies, deep learning may be able to detect subtle movement patterns that better predict AD biomarkers from video footage of finger‐tapping. We will also aim to improve test completion rates by conducting usability surveys and making refinements to instructions and the test setup.

## 5 CONCLUSION

This study shows that the brief webcam‐based finger‐tapping test, TAS Test, offers a new method to remotely aid detection of cognitive function in a community cohort and could be offered at significant scale. In this research, we look beyond the current definition of dementia – a clinical syndrome of cognitive decline – to investigate its early detection from a new perspective: by focusing on hand movement analysis. TAS Test requires refinement to ensure more reliable data capture, but such a simple test that easily crosses the usual language, cultural, and geographical barriers of standard cognitive tests, holds potential as a low‐cost population‐level tool to aid detection of subtle cognitive impairment in adults who do not have any overt cognitive decline.

## CONFLICTS OF INTEREST STATEMENT

Author disclosures are available in the supporting information.

## CONSENT STATEMENT

All human subjects provided informed consent.

## Supporting information



Supporting Information

Supporting Information

## Data Availability

The data supporting the findings of this study are available on request from the corresponding author. The data are not publicly available due to privacy or ethical restrictions.
