# Supplementary material for: Brief webcam test of hand movements predicts episodic memory, executive function, and working memory in a community sample of cognitively asymptomatic older adults
Source: Alzheimers Dement (Amst). 2024 Jan 25;16(1):e12520. doi: 10.1002/dad2.12520 (PMC10809289; doi:10.1002/dad2.12520)
Supplement: Supplementary file 1 — Supporting Information [file DAD2-16-e12520-s002.docx]

# Supplementary Materials

**S1. Random Forest regression**

| **Cognitive domain (test)** | **Hand** | **RMSE_best glm_** | **RMSE_RF_** |
| --- | --- | --- | --- |
| Episodic memory (PALTEA6) | Dominant | 4.1 | 4.2 |
|  | Nondominant | 4.0 | 4.2 |
| Executive function (SWMS) | Dominant | 2.6 | 5.6 |
|  | Nondominant | 2.6 | 5.5 |
| Working memory (SWMBE6) | Dominant | 3.2 | 4.4 |
|  | Nondominant | 3.2 | 4.4 |

**Table S1.1** Comparison of root mean squared error (RMSE) for best fitting GLM (regression) model reported in Results, and for a Random Forest regression model.


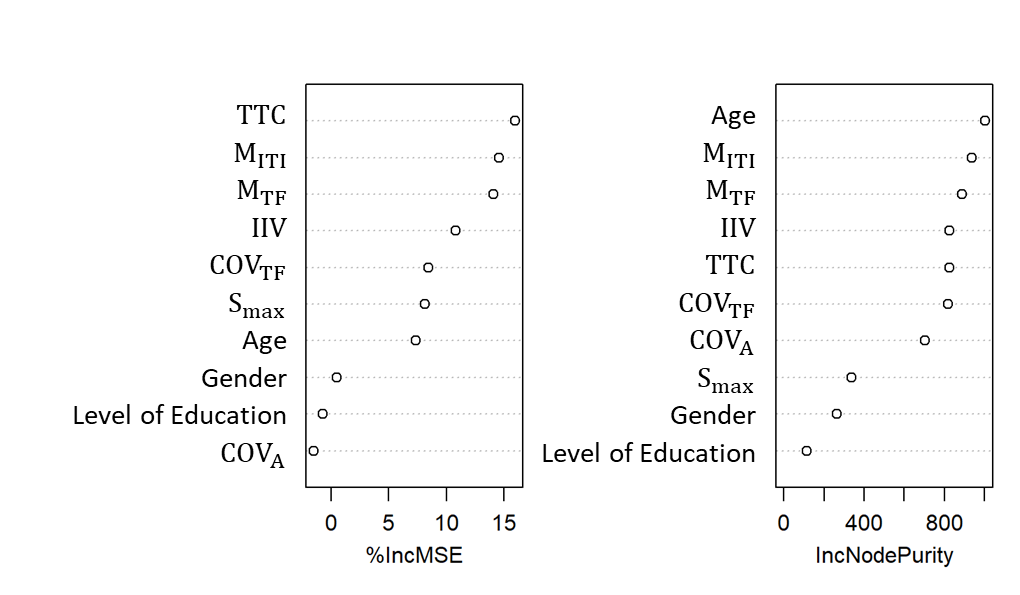


**Figure S1.1** Variable importance plots for Random Forest model regressing dominant hand finger-tapping features and demographic variables against episodic memory scores (PALTEA6). %IncMSE is percentage increase in mean squared error, and IncNodePurity is increase in node purity**.** Features are defined in the main document 2.2.3 Movement data extraction from finger-tapping videos.


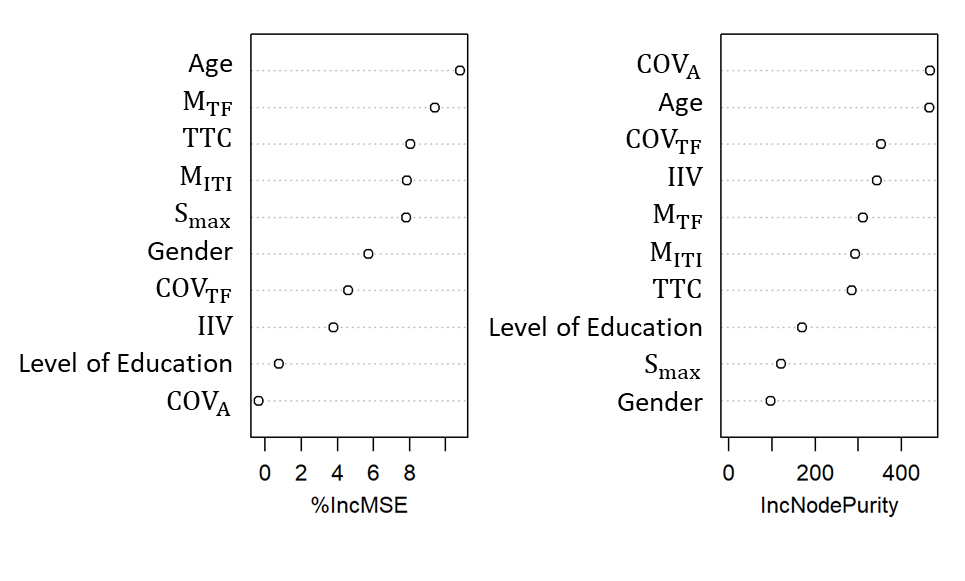


**Figure S1.2** Variable importance plots for Random Forest model regressing dominant hand finger tapping features and demographic variables against executive function scores (SWMS). %IncMSE is percentage increase in mean squared error, and IncNodePurity is increase in node purity**.** Features are defined in the main document 2.2.3 Movement data extraction from finger-tapping videos.


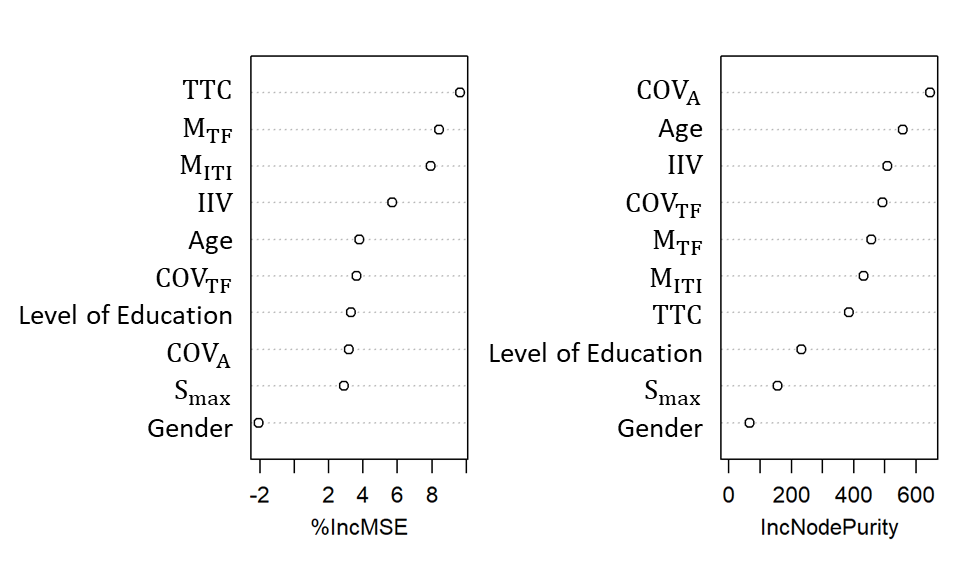


**Figure S1.3** Variable importance plots for Random Forest model regressing dominant hand finger-tapping features and demographic variables against working memory scores (SWMBE6). %IncMSE is percentage increase in mean squared error, and IncNodePurity is increase in node purity. Features are defined in the main document 2.2.3 Movement data extraction from finger-tapping videos.

**S2. Variance inflation factors (dominant hand finger-tapping features).**

| **Variable** | **VIF** | **Interpretation** |
| --- | --- | --- |
| Age | 1.088901 | Low |
| Gender | 1.087281 | low |
| Level of Education | 1.173019 | Low |
| $M_{\mathrm{TF}}$ | 7.721486 | Moderate |
| $\mathrm{COV}_{\mathrm{TF}}$ | 7.128734 | Moderate |
| $\mathrm{COV}_{A}$ | 1.312776 | Low |
| $\mathrm{TTC}$ | 5.362883 | Moderate |
| $M_{\mathrm{ITI}}$ | 2.629232 | Low |
| $\mathrm{IIV}$ | 3.344719 | Low |
| $S_{\max}$ | 6.707019 | Moderate |

**S3. Tables of coefficients for dominant hand finger-tapping features** ($\text{S}_{\text{MAX}}$ is maximum speed, $\text{M}_{\text{ITI}}$ is mean inter tap interval and TTC is total tapping count.)

|  | **PALTE6 (total errors** | | | **SWMS (strategy)** | | | **SWMBE6 (between errors** | | |
| --- | --- | --- | --- | --- | --- | --- | --- | --- | --- |
|  | **6-pattern adjusted)** | | |  |  |  | **6-pattern)** | | |
| *Predictors* | *Incidence Rate Ratios* | *CI* | *p* | *Incidence Rate Ratios* | *CI* | *p* | *Incidence Rate Ratios* | *CI* | *p* |
| (Intercept) | 5.11 | 2.89 – 9.49 | **<0.001** | 11.86 | 8.25 – 17.12 | **<0.001** | 15.26 | 2.97 – 93.24 | **0.001** |
| Age | 1.04 | 1.02 – 1.05 | **<0.001** | 1.01 | 1.01 – 1.02 | **<0.001** | 1.03 | 1.01 – 1.05 | **0.014** |
| Gender: Male | 1.14 | 0.90 – 1.46 | 0.278 | 0.87 | 0.80 – 0.94 | **0.001** | 1.02 | 0.74 – 1.43 | 0.893 |
| Left formal education before 16 years old | 1.47 | 0.74 – 2.85 | 0.259 | 1.06 | 0.84 – 1.33 | 0.637 | 1.91 | 0.73 – 4.72 | 0.171 |
| Left formal education at age 16 | 1.15 | 0.60 – 2.12 | 0.652 | 1.08 | 0.88 – 1.34 | 0.479 | 1.53 | 0.61 – 3.55 | 0.338 |
| Left formal education at age 17-18 | 1.12 | 0.61 – 1.93 | 0.703 | 1.02 | 0.84 – 1.24 | 0.872 | 1.26 | 0.53 – 2.67 | 0.566 |
| Undergraduate degree or equivalent | 0.97 | 0.52 – 1.74 | 0.926 | 1.09 | 0.90 – 1.35 | 0.384 | 1.59 | 0.65 – 3.51 | 0.28 |
| Master’s degree or equivalent | 0.65 | 0.32 – 1.30 | 0.226 | 0.94 | 0.74 – 1.19 | 0.606 | 0.7 | 0.25 – 1.82 | 0.47 |
| $\text{S}_{\text{MAX}}$ | 0.93 | 0.89 – 0.98 | **0.004** |  |  |  |  |  |  |
| $\text{M}_{\text{ITI}}$ |  |  |  | 0.62 | 0.40 – 0.91 | **0.024** | 0.1 | 0.01 – 0.87 | **0.027** |
| TTC |  |  |  | 0.99 | 0.98 – 1.00 | **0.003** | 0.96 | 0.93 – 0.99 | **0.004** |
| Observations | 404 | | | 404 | | | 404 | | |
| R^2^ Nagelkerke | 0.128 | | | 0.172 | | | 0.074 | | |
